# Predictors of immunization coverage among 12–23 month old children in Ethiopia: systematic review and meta-analysis

**DOI:** 10.1186/s12889-020-09890-0

**Published:** 2020-11-26

**Authors:** Tahir Yousuf Nour, Alinoor Mohamed Farah, Omer Moelin Ali, Mohamed Omar Osman, Mowlid Akil Aden, Kalkidan Hassen Abate

**Affiliations:** 1 Department of public health, College of Medicine and Health Sciences, Jigjiga University, P.O. BOX 1020, Jigjiga, Ethiopia; 2 Department of Population and Family Health, Jimma University, Jimma, Ethiopia

**Keywords:** Systematic review, Meta-analysis, Ethiopia, Predictors of immunization coverage

## Abstract

**Background:**

Immunization is one of modern medicine’s greatest achievements in the last three decades. Annually it can prevent nearly 2 to 3 million deaths. Understanding the determinants of effective immunization coverage is a critical undertaking. Accordingly, we set out to check the best available evidence of outstanding predictors of immunization coverage among children aged 12–23 months in Ethiopia.

**Method:**

Electronic databases including PubMed, Google Scholar, HINARI, and SCOPUS, Web of Science, African Journals Online, Ethiopian Medical Journals were searched. The search process, study selection, critical appraisal, and data extraction were done independently by two reviewers using Joanna Briggs Institute Meta-analysis for Review Instrument (JBI-MAStARI). The difference between reviewers was resolved with a third person. The risk of bias was assessed by the Newcastle Ottawa Tool for observational studies. Data were extracted using the Microsoft Excel checklist and exported to STATA 13. Heterogeneity was assessed using I^2^, Funnel plot and Egger’s test was used to check for publication bias**.**

**Results:**

We identified 26 studies with 15,042 children with mothers/caretakers to assess factors associated with immunization coverage and significant factors were: maternal formal education, (OR = 2.45; 95% CI: 1.62–3.72), paternal formal education, (OR = 1.01; 95% CI: 0.27–3.77), residence, (OR = 2.11; 95% CI: 1.00–4.45), birth at health facility (OR = 1.86; 95% CI: 0.99–3.49), family size less than four, (OR = 1.81; 95% CI: 1.16–2.84), knowledge on age of immunization to be completed (OR = 6.18;95% CI: 3.07–12.43), knowledge on immunization schedule (OR = 2.49; 95% CI: 1.35–4.59), time to travel to health faculties, (OR = 1.74; 95% CI: 0.62–4.89), antennal care, (OR = 3.11; 95% CI: 1.64–5.88), and tetanus toxoid vaccination, (OR = 4.82; 95% CI: 2.99–7.75).

**Conclusion:**

Our findings showed that literacy, residence, awareness, family size, maternal health services use, and proximity of the health facilities were factors associated with full immunization. This implies that there is a need for primary health service expansion and health education to “hard to reach areas” to improve immunization coverage for children aged 12–23 months.

## Background

Immunization is a proven tool for controlling and eliminating life-threatening vaccine-preventable diseases and it averts 2 to 3 million deaths annually [1]. World Health Organization (WHO) launched an expanded program of immunization (EPI) in 1974 to all its members. The program aimed to eradicate vaccine-preventable diseases (VPDs) such as: diphtheria, measles, pertussis, tetanus, poliovirus, and tuberculosis [2].

A recent WHO report found that 13.5 million infants had not received a vaccination dose of any kind, 19.4 million did not recieve the third dose of DPT, and 12 million unvaccinated children lived in just ten countries: Angola, Brazil, the Democratic Republic of the Congo, Ethiopia, India, Indonesia, Nigeria, Pakistan, the Philippines, and Vietnam [3, 4].

Globally, from 1990 to 2018 under-five deaths ranged from 96 to 41 per 1000 live births. In the same years, Sub-Saharan Africa (SSA) ranged from (189 to 83/1000 live births). In 2018, under-five mortality in Ethiopia was (61 per 1000 live births) which is higher than most SSA countries [5].

The most common leading causes of under-five deaths are pneumonia, diarrhea, measles, and neonatal conditions that can be prevented or treated with simple affordable intervention such as vaccination or antibiotics [6]. Annually, 3.37 million children acquire pneumonia worldwide with an estimated 1.4 million or 18% of all under-five children deaths. The majority of children who acquired pneumonia live in South Asia and Sub-Saharan Africa [7]. In Ethiopia, 6.6% of children under 5 had experienced symptoms of Acute Respiratory Infection (considered a proxy for pneumonia) and of those with the symptoms, treatment from a health facility or provider was sought for 31.6% only [8].

Universally, around 1.7 million cases of diarrhea occur annually [9]. In East African countries alone 13–32% of under-five mortality is due to diarrhea [10]. Only one in three children with episodes of diarrhea receive oral rehydration salts while less than 5% are receiving zinc supplement for diarrhea treatment [11]. Similarly, in Ethiopia 11.8% of under-five children experienced diarrhea and only 44.6% of children sought treatment from a health facility or health service provider. Among those children that sought treatment, 38% of children with diarrhea reportedly received rehydration solution in a form of an ORS sachet or a recommended home fluid [8].

Worldwide, 7 million people are affected by measles. Among the measles cases, 15% are severely complicated while only one-third of them received medical attention [12]. Likewise, measles incidence in Ethiopia is 50 cases per million per year which is way beyond the Ethiopian national measles elimination plan by the year 2020. The case fatality rate is 3 to 6% which is even underestimated due to incomplete reports [13].

Both community and morbidity based reports revealed that VPDs are public health problems in Ethiopia [14–16]. In Ethiopia, EPI was started in 1980 with aim of reducing maternal and child morbidity and mortality from VPDs [17]. EPI is provided through static, outreach, and mobile services with an immunization schedule of a dose bacillus Calmette–Guerin (BCG) and oral polio vaccine first dose (OPV0). Then three doses of OPV, three Penta-valent, two doses of Rota, and three pneumococcus vaccines which are given at 4th, 6th, 10th, and 14th weeks, respectively. Finally, the measles vaccine is given at the age of 9 months [18]. Recently, the second dose of measles vaccine was introduced to the routine immunization schedule and given at 15 months old children [13].

Ethiopian government has brought maternal, newborn, and child health as priority political agenda and maintained its commitment to improving the health and survival of women and children in the country (18). This has been demonstrated by massively expanding access to and utilization of key health care services through the Health Extension Program (HEP) (Tekelab et al., 2019). Health service delivery thus decentralized to nine regional states and two city councils, from zonal to Keble level (smallest administrative unit), with vaccines being supplied by the Federal Ministry of Health (FMOH) and developmental partners like GAVI and UNICEF, whom are also providing technical support to the government [18].

Although some studies reported determinants of immunization coverage in Ethiopia, they are not consistent and varied across the country. The reported determinants include ANC and PNC coverage, knowledge on age of a child to be vaccinated, parents formal education, place of delivery, residence, traveling time to health facilities, TT vaccination, family size, household wealth status, and marital status [19–23]. Thus, the current work aims at identifying relevant studies and summarizing major predictors of immunization coverage in Ethiopia. The results of this review will add to existing knowledge of the problem, and guide policymakers to improve immunization programs in Ethiopia.

## Method

### Protocol and registration

This systematic review and meta-analysis of predictors (factors) of immunization coverage among 12–23 month old children in Ethiopia was registered with international prospective register and systematic reviews PROSPERO 2020 RD42020166791. Available at: https://www.crd.york.ac.uk/prospero/display_record.php?ID=CRD42020166791.

### Searching strategy

We performed electronic searches of articles included in this systematic review from PubMed, Google Scholar, EMBASE, HINARI, SCOPUS, Web science, African Journals Online databases, and Ethiopian Medical Journal of which are all open sources. All relevant articles were also searched and retrieved manually from already identified articles’ references. In addition, a Grey literature search was also done.

### Study selection

The Preferred Systematic Reviews and Meta-Analysis (PRISMA) checklist was used in the formulation of systematic literature reviews. The reviewers searched articles from electronic databases based on their titles and abstracts. We selected studies assessed predictors of immunization coverage, Full text of selected studies reviewed, critical appraised, and data extracted. These search terms were pre-defined to allow a comprehensive search strategy that included all fields within records and Medical Subject Headings (MeSH) to expand the search in an advanced PubMed search as showed in Appendix I (Additional files 1).

### Inclusion and exclusion criteria

#### Study design and period

observational study design (cohort, cross-sectional, and case-control) those reported predictors of immunization coverage among 12–23-month-old children published from 2009 were included.

#### Study setting

Both community and facility-based observational study designs were included.

#### Population/participant

Study participants were children aged 12–23 months.

Language: Studies reported in the English language only were considered to be eligible for this systematic review and meta-analysis.

#### Publication condition

Both published and unpublished articles were considered.

#### Exposure

Factors or determinants of immunization coverage in Ethiopia.

#### Outcome

Children aged 12–23 months who those are fully vaccinated.

### Exclusion

After we have examined eligibility criteria of both published and unpublished studies, articles didn’t fulfil inclusion criteria or full text were not got after solo authors contacted two times were excluded from this systematic review and meta-analysis. We also excluded case studies, reviews, theses, letters to editors, editorials, commentaries, and conference abstracts.

### Selection criteria

#### Measurement of the outcome variable

The aim of this review is to assess predictors of immunization coverage among 12–23 months old children in Ethiopia. As per WHO definition, children are considered fully vaccinated when they receive one dose of Bacillus Calmette Guerin (BCG), three doses of DPT, three doses of Oral Polio Vaccine (OPV), and one dose of Measles Conjugated Vaccine (MCV) at the age of 9 to 12 months [24].

Identified predictors were maternal formal education, (formal education versus non-formal education), paternal formal education, (formal education and non-formal education), residence (urban versus rural), place of delivery (health facility versus home), family size (< 4 and > = 4), maternal knowledge on age to vaccinate their children (good versus poor), maternal knowledge on schedule (good versus verses poor), knowledge on benefit of vaccination (good versus poor), travelling time to health facility (< = 60 min and > 60 min), antenatal care follow-up (yes versus no), postnatal care (yes versus no) and maternal TT vaccination (yes versus no).

#### Data extraction procedures

Two authors (TY and AM), independently extracted all necessary data based on pre-defined inclusion criteria using checklist ready from Microsoft Excel Sheet. Data extraction format consists: author, year of publication, region, study area, study design, sample size of determinant factors. Any disagreement between two authors was resolved through consensus between them, and a third reviewer (KHA) was asked to solve any disagreement that occurred. Data extraction format prepared was a two by two table in Appendex II (Additional file 2), log OR was calculated for each factor based on a two by two table from original studies.

#### Quality assessment

Two independent reviewers critically appraised articles with standardized critical appraisal format from Joanna Briggs Institute Meta-analysis for Review Instrument (JBI-MAStARI), Joanna Briggs Institute, University of Adelaide, Australia for observational studies (applied cross-sectional and case-control study designs). The checklist has eight questions for analytical cross-sectional and ten for case-control study designs with four possible answered (I. Yes, II. No, III. Unclear, IV. Not applicable) as shown (Appendix III) (Appendix IV). Three for cross-sectional studies i) selection of study design (four points). ii) comparability of study design (two points). iii) Outcome (three points). Three components for case-control i) selection of study (four points). ii) comparability of study design (two points). iii) ascertainment of exposure (three points) as showed (Additional files 3 and 4) respectively [25, 26]. Two reviewers also checked the quality of primary articles using Newcastle-Ottawa Quality Assessment Scale for observational studies (Additional file 5). Articles with methodological problems, incomplete, full texts not available were excluded from the final analysis.

#### Data synthesis

Data synthesis was carried out using STATA version 13. Forest plot was run and presented Odd Ratio (OR) with 95% CI, *P*-value and I^2^. Heterogeneity was assessed based on (Cochrane handbook) with a cutoff point of I^2^ 0–40, 30 to 60%, 50 to 90%, 75 to 100% declared heterogeneity as: might not be important, moderate, substantial, and considerable heterogeneity. The funnel plot is the first step to assess the presence of publication bias and as a rule of thumb it is used only when meta-analysis contains ten and more studies [27]. Then Eggers’ regression test was done to decide the presence of publication bias when *p*-value < 0.10 [28]. This study was also reported based on the PRISMA flow diagram [29].

## Results

**Fig. 1:**
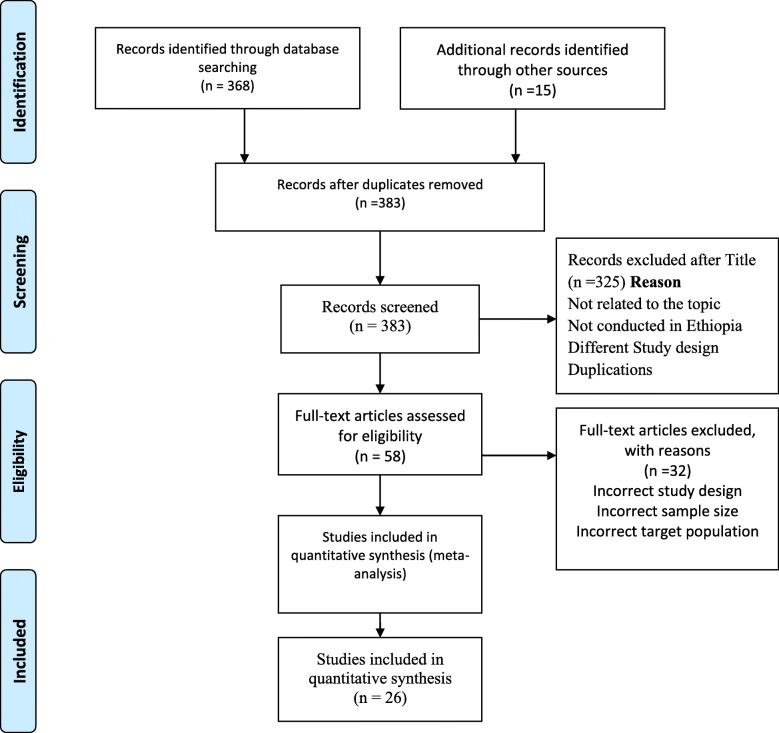
Flow chart explaining selection of primary studies

In the first step, we searched, reviewed, and retrieved 383 published and unpublished articles from electronic databases. All articles were imported into EndNote software (version X7; Thomson Reuters, New York, NY) and 34 articles were excluded due to duplication. In addition, 291 articles were excluded for not relating to the topic, not done in Ethiopia, study design, and inconsistency with inclusion criteria set by the reviewers. Furthermore, 32 articles were excluded due to incorrect sample size, study design, and target. Finally, 26 articles were eligible and included in this meta-analysis as shown in the PRISMA follow chart (Fig. 1).

### Characteristics of the included studies

All 26 included articles were observational studies (cross-sectional and case-control) with a total participant of 15,042 (Table 1). Their sample size ranged from 266 for Wonago district of the South region [37] to 1534 for Amibara woreda (district) of the Afar region [42]. Based on geographical distribution of the 26 included studies, eight studies were conducted in Amhara region [20, 22, 30, 32, 38, 43, 49, 50], nine in SNNP region [23, 33, 34, 36, 37, 40, 41, 46], four in Oromia region [21, 39, 48, 51], two from Tigray region [31, 35] one in Afar region [42], one in Somali region [44] and one from pastoral/Semi-pastoral regions (Benishangul Gumuz, Gambella, Oromia, Somali, and Southern Nations, Nationalities and Peoples’ regions) [47], and all were above cutoff points of 50%. Based on factors associated with immunization coverage there were 12 selected factors and synthesized from identified eligible as shown (Table 2).

**Table 1:** Characteristics of included observational studies in this systematic review and meta-analysis in Ethiopia

Author	Region	Study setting	Study area	Study design	Sample size	Response rate	NOS	Study subject	Predictors OR 95% CI
Animaw W et al. 2014 [30]	Amhara	community based	Arba Minch town and Arba Minch Zuria District	CS	630	100%	7	12–23 month	Mother had formal education, (OR = 4.60; 95% CI: 3.13–6.77), gave birth to the health facility, (OR = 3.20; 95% CI: 2.00–5.15), knowledge on immunization schedule (OR = 0.46; 95% CI: 0.30,0.71).travelling time < 60 min, (OR = 7.60; 95% CI: 2.67–21.66)
Aregawi G et al. 2017 [31]	Tigray	community based	Laelay Adiabo District	UMCC	270	100%	8	12–23 month	Knowledge on schedule,(O*R* = 6.98; 95% CI: 3.94–12.37), PNC follow up, (OR = 7.08; 95% CI: 3.67–13.65)
Asfaw A et al. 2019 [23]	SNNP	community based	Sodo Zurea Distric	UMCC	344	100%	9	12–23 month	Had formal education, (OR = 2.34;95% CI: 1.15–3.64), knowledge on immunization schedule, (OR = 2.79, 95% CI: 1.73–4.48), PNC follow up, (OR = 2.69; 95% CI: 1.73–4.48)
Ayal D and Bekele T 2014 [32]	Amhara	community based	Mecha District	CS	497	100%	9	12–23 month	Father had formal education, (OR = 1.28, 95% CI: 0.90,1.82), urban residence, (OR = 3.94, 95% CI: 1.76–8.82). gave birth to the health facility, (OR = 1.92, 95% CI: 1.30,2.84), knowledge on immunization schedule, (OR = 2.77, 95% CI: 1.42–5.39)
Birhan, Y., et al. 2014 [33]	SNNP	community based	Hawassa Zuria District	UMCC	308	98%	8	12–23 month	Mother had formal education,(0R = 5.62;95% CI: 3.34–9.47), urban residence (OR = 0.34, 95% CI: 0.17–0.69), gave birth to the health facility (OR = 5.95;95% CI: 2.93,12.11). knowledge on immunization -schedule,(O*R* = 5.02; 95% CI: 2.79–9.04) knowledge on immunization benefit, (OR = 2.05;95% CI: 1.09–3.87). ANC follow up, (OR = 0.98;95% CI: 0.65,1.49). TT vaccination, (OR = 3.47;95% CI: 2.10–5.73).
Bizuneh Ayano 2014 [34]	SNNP	community based	Hosanna Town	CS	508	100%	8	12–23 month	knowledge on immunization schedule, (OR = 2.28;95% CI:1.79–9.04). ANC follow up, (OR = 6.49; 95% CI: 2.76–15.26). TT vaccination, (OR = 4.85;95% CI: 3.38–6.97)
Etana and Deressa 2012 [21]	Oromia	community based	Ambo District	CS	536	100%	7	12–23 month	Gave birth to the health facility, (OR = 0.71;95%CI: 0.43,1.17). knowledge on age immunization complete, (OR = 10.03; 95% CI: 5.02,9.75). ANC follow up, (OR = 6.38; 95% CI: 4.01,10.15)
Girmay and Dadi 2019 [35]	Tigray	community based	Debre Markos Town	CS	620	99.5%	8	12–23 month	Mother had formal education, (OR = 1.77; 95% CI: 1.20–2.59). gave birth to the health facility, (OR = 3.52;95% CI: 2.36–5.25), family size > = 4, (OR = 2.34; 95% CI: 1.54,3.57). knowledge on immunization schedule, (OR = 0.22; 95% CI: 0.15–0.34), travelling time < 60 min, (OR = 2.20; 95% CI: 1.47–3.30)
Gualu and Dilia 2017 [22]	Amhara	community based	Debre Markos Town	CS	288	96.8%	7	12–23 month	ANC follow up, (OR = 3.67; 95% CI: 1.32–10.19)
Hailu et al. 2019 [36]	SNNP	community based	Wonago district	CS	1116	82.70%	7	12–23 month	Knowledge on immunization schedule, (OR = 2.57;95% CI: 2.00–3.30). ANC follow up, (OR = 0.61;95% CI: 0.46–0.80), TT vaccination, (OR = 3.37;95% CI: 2.58–4.40)
Henok T et al. 2009 [37]	SNNP	community based	Wonago district	UMCC	266	99.2%	7	12–23 month	Knowledge on immunization benefit, (OR = 2.58;95% CI: 1.09–6.15).
Kassahun et al. 2015 [38]	Amhara	community based	Lay Armacheho district	CS	751	99.2%	8	12–23 month	Knowledge on age immunization complete, (OR = 2.42,95% CI: 1.71–3.43), urban residence, (OR = 1.47;95% CI: 0.96–2.26). TT vaccination (OR = 2.45;95% CI: 1.64–3.64)
Lake et al. 2014 [36]	Amhra	community based	Dassie town	CS	724	100%	7	12–23 month	Knowledge on immunization schedule, (OR = 6.12, 95% CI: 4.41,8.49). knowledge on age immunization complete, (OR = 0.56; 95% CI: 0.24–1.29) family size > = 4, (OR = 1.14;95% CI: 0.75–1.72)
Legesse and Dechasa 2015 [39]	Oromia	community based	Sinana district	CS	519	98.5%	8	12–23 month	Knowledge on immunization schedule, (OR = 2.16;95% CI: 1.45–3.22), father had formal education, (OR = 0.18;95% CI: 0.07–0.44), travelling time < 60 min, (OR = 2.16;95% CI: 0.69–6.72), ANC follow up, (OR = 3.03,95% CI: 2.02–4.55)
Melaku Kindie et al. 2018 [40]	SNNP	community based	Amanuel district	UMCC	308	100%	9	12–23 month	gave birth to the health facility, (OR = 0.15;95% CI: 0.09–0.25), ANC follow up, (OR = 5.10;95% CI: 3.12–8.34), PNC follow up, (OR = 3.28;95% CI: 1.84–5.85).
Meleko et al. 2018 [41]	SNNP	community based	Mizan Aman town	CC	322	100%	8	12–23 month	Mother had formal education, (OR = 3.03;95% CI: 1.91–4.80), gave birth to the health facility, (OR = 2.05;95% CI: 1.30–3.23)
Mebrahtom and Birhane 2013 [42]	Afar	community based	Amibara district	CS	1534	98.3%	8	12–23 month	Knowledge on immunization schedule, 21.50 (11.47–40.30). ANC follow up, (OR = 20.49;95% CI: 10.32–40.70), PNC follow up, (OR = 4.07;95% CI: 2.67,6.22). TT vaccination, (OR = 36.80;95% CI: 14.95–90.56)
Mekonnen et al. 2019 [43]	Amhara	community based	Minjarshenkora district	CS	566	98.8%	8	12–23 month	Travelling time < 60 min, (OR = 1.95; 95% CI: 1.30–2.92)
Mohammed and Atomsa 203	Oromia	community based	Kombolcha District	CS	685	98.7%	7	12–23 month	ANC follow up, (OR = 2.42;95% CI: 1.64–3.58)
Mohamud et al. 2014 [44]	Somali	community based	Jigjig district	CS	582	100%	8	12–23 month	Mother had formal education, (OR = 3.63;95% CI: 2.17–6.08), gave birth to the health facility, (OR = 3.06;95%CI: 2.10–4.47), urban residence, (OR = 2.63;95% CI: 1.85–3.73), TT vaccination, (OR = 4.94;95% CI: 2.82–8.66)
Negussie A et al. 2016 [45]	SNNP	community based	Arbegona district	CS	548	99.45%	7	12–23 month	Knowledge on immunization benefit, (OR = 6.30;95% CI: 2.33–3.87). family size > = 4, (OR = 2.22; 95% CI: 1.52–3.24)
Tefera et al. 2018 [46]	SNNP	community based	Worabe town	CS	484	89.6%	8	12–23 month	travelling time < 60 min, (OR = 0.30;95% CI: 0.20–0.44), ANC follow up, (OR = 0.32;95% CI: 0.11–0.95)
Tesfaye et al. 2018 [20]	Amhara	community based	East Gojam Zone	CS	846	98.1%	8	12–23 month	Urban Residence, (OR = 1.63; 95% CI: 1.23–2.15), ANC follow up, (OR = 3.86;95% CI: 2.42–6.16)
Tessema et al. 2019 [47]	Pastoral/semi-pastoral	community based	CORE Group Polio Project implementation areas	CS	600	96.6%	8	12–23 month	Urban residence, (OR = 9.39;95% CI: 6.42–13.75)
Wado et al. 2014 [48]	Oromia	community based	Gilgel Gibe Health and Demographic Surveillance System	CS	889	100%	8	12–23 month	Mother had formal education, (OR = 1.46;95% CI: 1.07–1.99)
Yismaw A et al. 2019 [49]	Amhara	community based	Gondar city	CS	301	100%	8	12–23 month	Knowledge on age immunization complete,(O*R* = 9.43;95% CI: 5.16–17.23), knowledge on immunization benefit, (OR = 1.54;95% CI: 0.82–2.89)

Key: *CS* cross-sectional, *UMCC* unmatched case-control

**Table 2:** Meta-analysis summary of included and synthesised predictors on immunization coverage in Ethiopia

s.no	Factors /authors	Number of study	OR, 95% CI	*P*-value	I^2^	Egger’s test (*p*-value)
1	Mother with formal education [23, 30, 33, 35, 41, 44, 48, 50]	8	(OR = 2.45;95% CI: 1.62–3.72)	0.000	85.6%	0.883
2	Father with formal education [32, 39, 41]	3	(OR = 1.01;95% CI: 0.27–0.77)	0.000	94.5%	0.675
3	Residence [20, 32, 33, 38, 44, 47]	6	(OR = 2.11;95% CI: 1.00–4.45)	0.000	94.6%	0.784
4	Place of delivery (health facility) [20, 21, 30, 33–35, 40, 41, 44]	9	(OR = 1.86;95% CI: 0.99–3.49)	0.000	94.6%	0.428
5	Family size > = 4 [35, 45, 50]	3	(OR = 1.81;95% CI: 1.16–2.84)	0.026	72.9%	0.767
6	knowledge on age to complete [21, 38, 49, 52]	4	(OR = 6.18;95% CI: 3.07–12.43)	0.000	90.2%	0.432
7	Knowledge on schedule [20, 23, 30–36, 39, 42, 50]	12	(OR = 2.49; 95% CI: 1.35–4.59)	0.000	96.2%	0.621
8	Knowledge on benefit of immunization [33, 37, 40, 49]	4	(0R = 2.43;95% CI: 1.44–4.09)	0.129	47.1%	0.091
9	Travelling time to health facilities > = 60 min [30, 35, 39, 43, 46, 53]	6	(OR = 1.76;95% CI: 0.62–4.89)	0.000	94.5%	0.535
10	ANC follow up [20–22, 33–36, 39, 40, 42, 46, 51]	12	(OR = 3.11;95% CI: 1.64–5.88)	0.000	95.0%	0.104
11	PNC follow up [23, 31, 40, 42]	4	(OR = 3.83;95% CI: 2.65–5.52)	0.105	51.2%	0.405
12	TT vaccination [33, 34, 36, 38, 42, 44]	5	(OR = 4.84;95% CI: 2.99–7.75)	0.000	85.8%	0.124

### Predictors of immunization coverage in Ethiopia

#### Socio-demographic factors

**Fig. 2:**
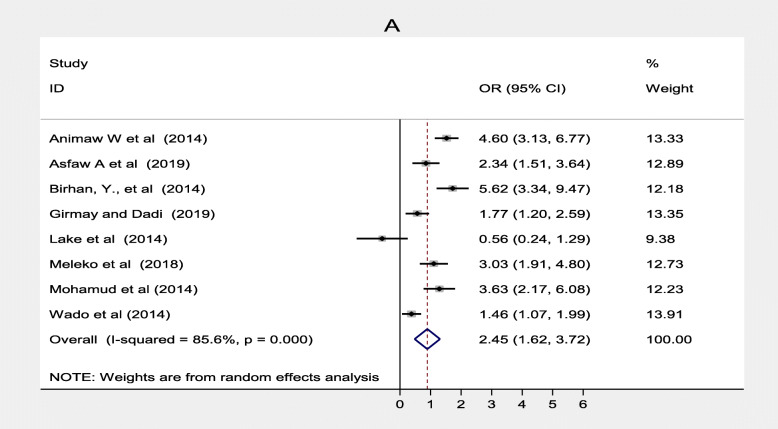
Forest plot depicting the association between mother with formal education and immunization coverage among 12–23 months old children in Ethiopia

The result of this review revealed that some socio-demographic factors associated with immunization coverage among 12–23-month-old children in Ethiopia. These factors were; mother’s educational status, father’s educational status, residence, place of delivery and family size. Eight studies [23, 30, 33, 35, 41, 44, 48, 50] indicated that women’s level of education was significantly associated with immunization coverage in Ethiopia. Women who had attended formal education were 2.45 times more likely to immunize their children compared to their counterpart (OR = 2.45; 95% CI: 1.62–3.72) (Fig. 2).

**Fig. 3:**
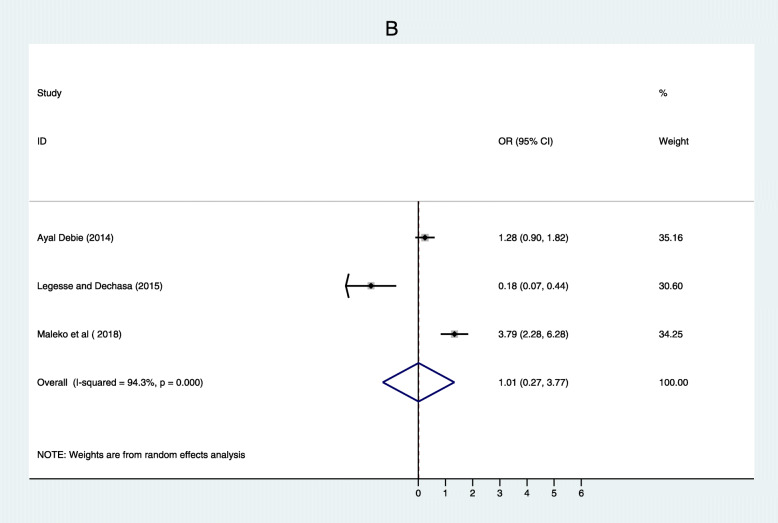
Forest plot depicting the association between Father with formal Education and immunization coverage among 12–23 months old children in Ethiopia

**Fig. 4:**
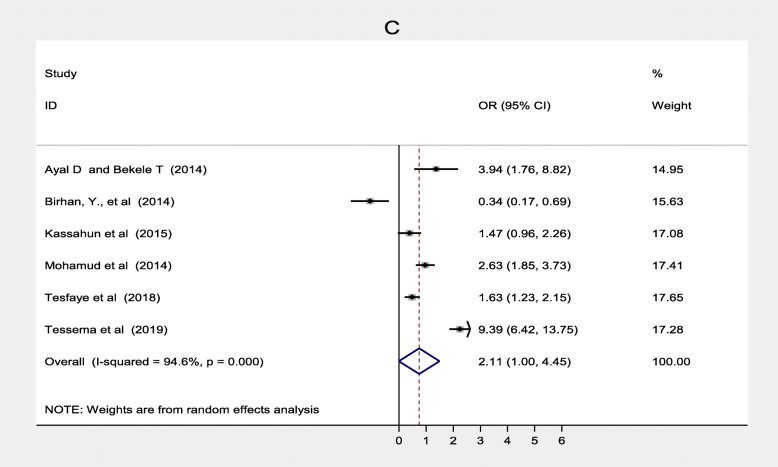
Forest plot depicting the association between residence and immunization coverage among 12–23 months old children in Ethiopia

Three studies [32, 39, 41] also found father’s educational status was associated with immunization coverage in Ethiopia. Children of fathers who had formal education were 1.01 more likely to complete routine vaccination than those who had non-formal education (OR = 0.01, 95% CI: 0.27–3.77). Heterogeneity test for both women’s and father’s educational status was substantial and considerable with I^2^ = 85.6 and 94.3% respectively. Both of them showed there was no publication bias based on Egger’s test with *p* = 0.883 and *p* = 0.675 respectively (Fig. 3). Six studies [20, 32, 33, 38, 44, 47] also showed that residence was significantly associated with immunization coverage in Ethiopia. This systematic review and meta-analysis revealed the rural residence was one of the determinants of immunization coverage. Women from rural residence were 1.58 times less likely to fully vaccinate their children compared to their counterparts (OR = 2.11; 95% CI: 1.00–4.45). There was no considerable heterogeneity among studies (I ^2^ = 94.6%) s while Egger’s test *p* = 0.784 showed no significant publication bias (Fig. 4).

**Fig. 5:**
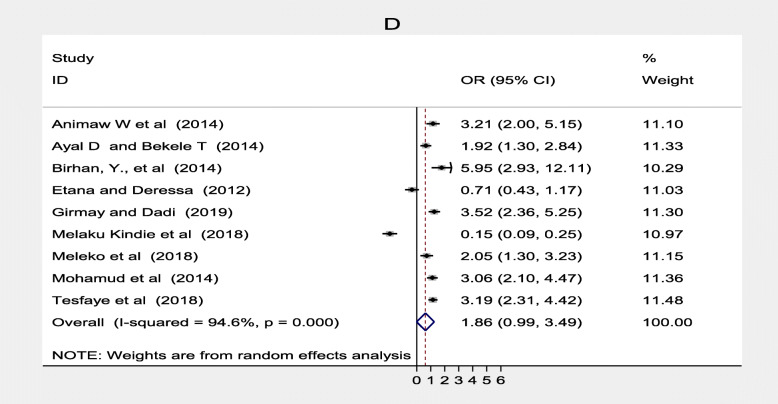
Forest plot depicting the association between place of delivery and immunization coverage among 12–23 months old children in Ethiopia

Similarly, nine studies [20, 21, 30, 33–35, 40, 41, 44] found that women who gave birth in the health facilities were 1.89 times more likely to complete routine immunization than those who gave birth at home (OR = 1.86; 95% CI: 1.35–3.49). The heterogeneity test indicated that there was a considerable heterogeneity assessed place of delivery (I^2^ = 94.6%) and no publication bias Egger’s test was *p* = 0.428 (Fig. 5).

**Fig. 6:**
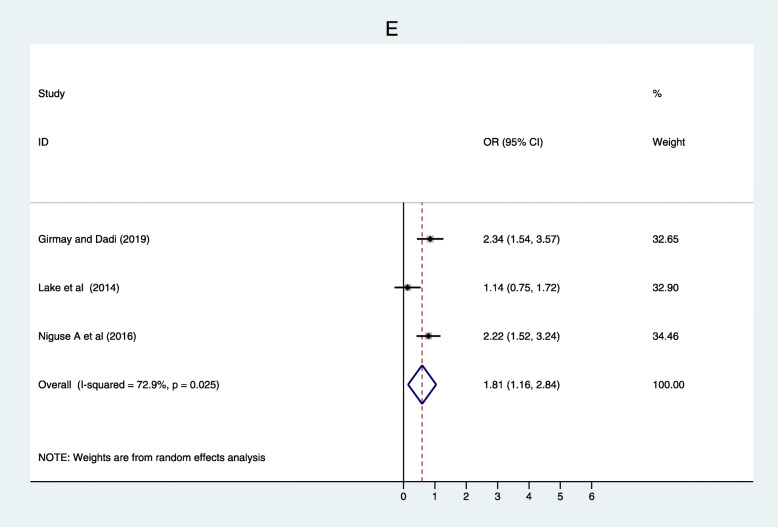
Forest plot depicting association between Family size and immunization coverage among 12–23 months old children in Ethiopia

Based on evidence from three studies [35, 45, 50] family size was associated with full vaccination. Households that had a family size less than four were more likely to complete immunization to their children compared to their counterpart (OR = 1.81; 95% CI: 1.16–2.84). There was a substantial heterogeneity test I^2^ = 72.9%, while Egger’s test *p* = 0.767 shows that there was no publication bias (Fig. 6).

#### Maternal and health facility related factors

Some maternal and health facility-related factors were also found to be significantly associated with immunization coverage. Maternal knowledge on age to complete immunization, knowledge on the immunization schedule, traveling time to the health facility was strongly associated with immunization coverage. Whereas, maternal knowledge on the benefit of immunization was not statistically significant to immunization coverage in Ethiopia.

**Fig. 7:**
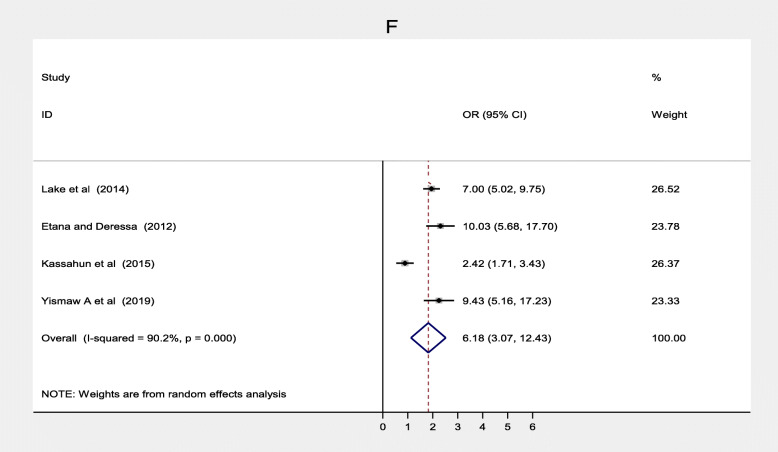
Forest plot depicting the association between knowledge on age to complete immunization and immunization coverage among 12–23 months old children in Ethiopia

Maternal knowledge on age to complete immunization was significantly associated with immunization coverage in Ethiopia [21, 38, 49, 52]. Women who had adequate knowledge on age to be completed routine immunization were more likely to vaccinate their children fully compared to women who had inadequate knowledge, (OR = 6.18; 95% CI: 3.07–12.43). The heterogeneity test indicated that there was a considerable significant heterogeneity (I^2^ = 90.2%), and Egger’s test found there was no publication bias (*p* = 0.432) (Fig. 7).

**Fig. 8:**
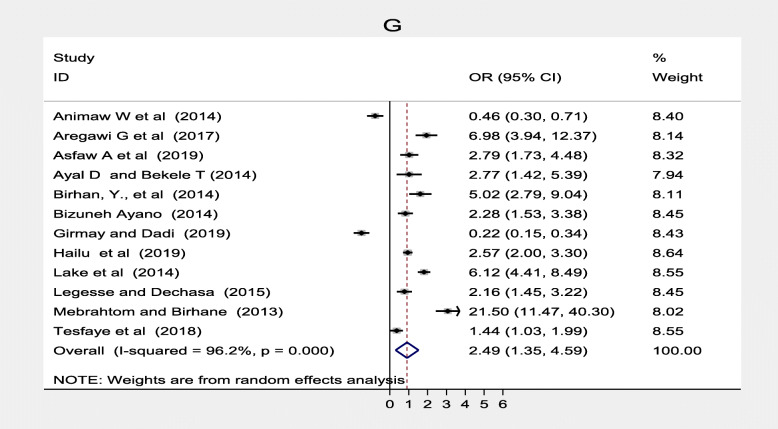
. Forest plot depicting the association between knowledge on immunization schedule and immunization coverage in Ethiopia

**Fig. 9:**
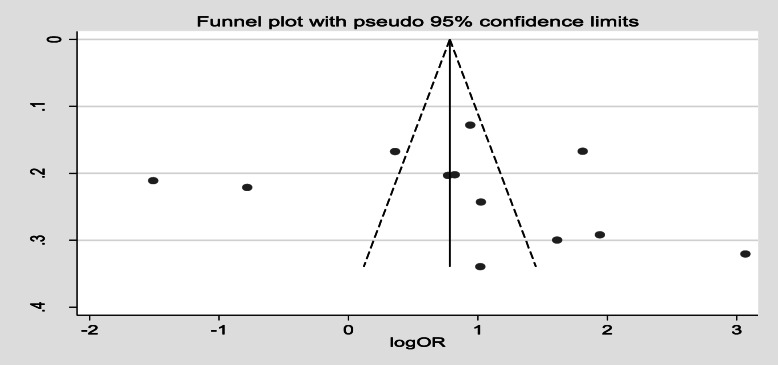
funnel plot of publication bias knowlege on immunization schedule among 12–23 months old children in Ethiopia

**Fig. 10:**
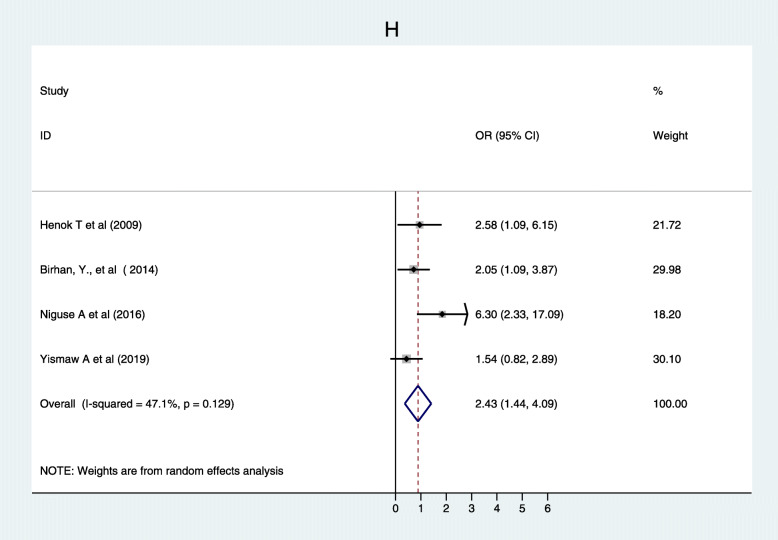
Forest plot depicting the association between knowledge benefit of immunization and immunization coverage among 12–23 months old children in Ethiopia

**Fig. 11:**
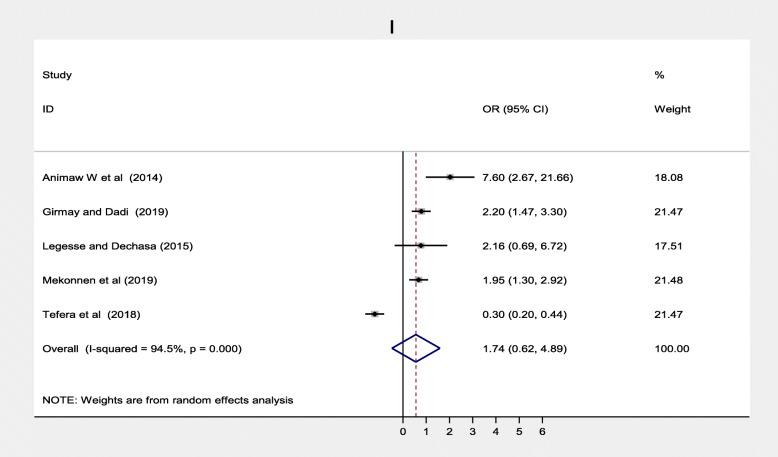
Forest plot depicting the association between traveling time to the health facility and immunization coverage among 12–23 months old children in Ethiopia

Furthermore, 12 studies [20, 23, 30–36, 39, 42, 50] showed maternal knowledge on immunization schedule was significantly associated with vaccination coverage in Ethiopia. Women who had good knowledge of immunization schedules were 2.49 times more likely to fully vaccinate their children compared to women who had poor knowledge of the immunization schedule, (OR, 2.49; 95% CI: 1.35–4.59). There was a considerable heterogeneity test among studies (I^2^ = 96.2%) as shown (Fig. 8). Funnel plot illustrated the presence of publication bias but Egger’s test revealed there was no publication bias (0.621) and we concluded that there was no publication bias as shown (Fig. 9). On the other hand, four studies [33, 37, 40, 49] revealed that there were no significant association between knowledge on the benefit of immunization and immunization coverage (Fig. 10). Six studies [30, 35, 39, 43, 46, 53] also indicated there was an association between traveling time to the health facility and immunization coverage. Women who had to walk for less than or equal to 60 min to the health facility were more likely to fully vaccinate their children compared to those who had more than a 1 h walk (OR, 2.33; 95% CI: 0.80–6.79). Heterogeneity test (I^2^ = 95.9%) showed there was no considerable heterogeneity. Based on Egger’s test weighted regression statistics (*p* = 0.535) there was no indicative publication bias (Fig. 11).

#### Maternal health care utilization factors

**Fig. 12:**
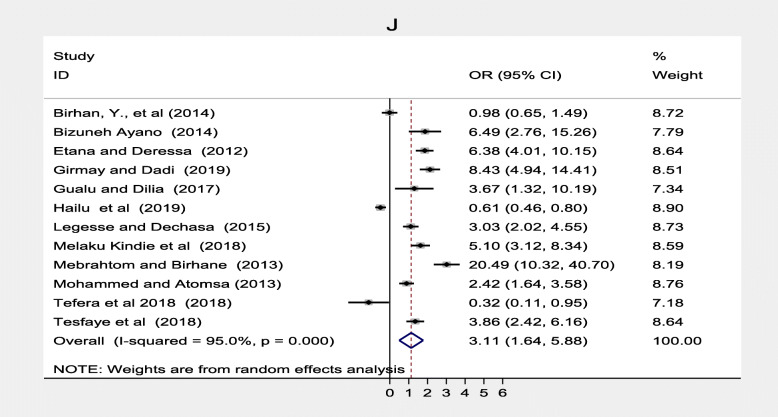
Forest plot depicting the association between ANC and immunization coverage among 12–23 months old children in Ethiopia

**Fig. 13:**
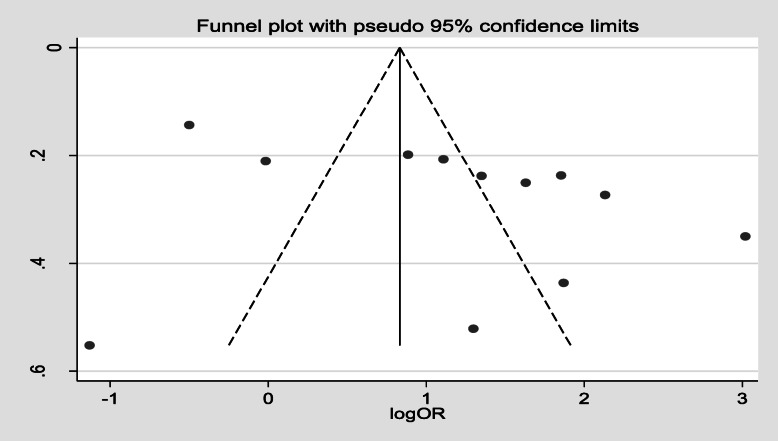
Funnel plot for publication bias of ANC in Ethiopia

**Fig. 14:**
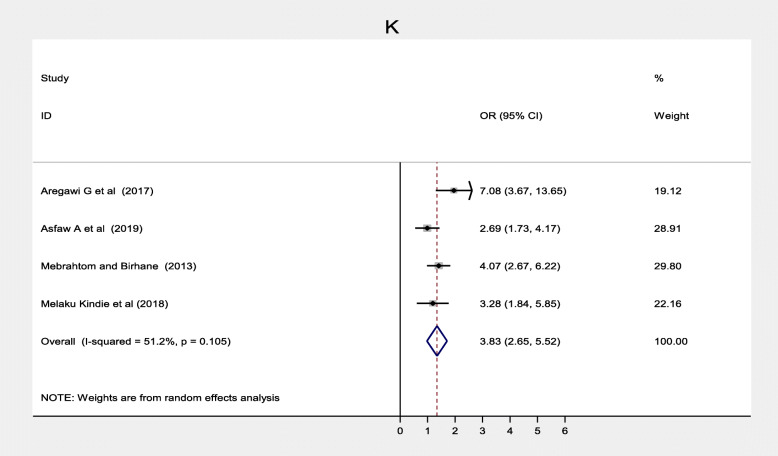
Forest plot depicting the association between PNC and immunization coverage among 12–23 month old children in Ethiopia

**Fig. 15:**
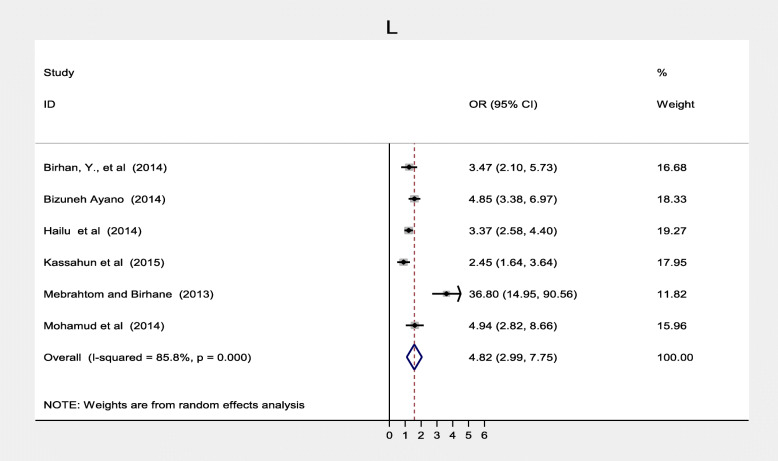
Forest plot depicting the association between TT vaccination and immunization coverage among 12–23 months old children in Ethiopia

Maternal health care utilization factors such as antenatal care, maternal TT vaccination was also significantly associated while postnatal care was not significantly associated with immunization coverage in Ethiopia. Twelve studies [20–22, 33–36, 39, 40, 42, 46, 51] found a significant association between antenatal care and full vaccination in Ethiopia. Women who had ANC follow up were three times more likely to complete their children’s vaccination program compared to women who followed ANC, (OR = 3.11; 95% CI: 1.64–5.88) (Fig. 12). With the random effect model I^2^ = 95.0% substantial heterogeneity was found. Funnel plot was asymmetrical which indicates the presence of publication bias while Egger’s test (*p* = 0.104) indicated the absence of publication bias, thus we concluded that there was no publication bias (Fig. 13). On the contrary, four studies [23, 31, 40, 42] revealed that there were no association between postnatal care and immunization coverage in Ethiopia, as it is depicted (Fig. 14). Six studies [33, 34, 36, 38, 42, 44] also indicated that TT vaccination was significantly associated with immunization coverage. Women who took TT vaccination during ANC follow-up were 4.82 times more likely to complete immunization of their children compared to those who had not taken TT vaccination (OR = 4.82; 95% CI: 2.99–7.75). I^2^ test = 85.5% showed that there was substantial heterogeneity while Egger’s test *p* = 0.124 depicts that there was no publication bias (Fig. 15) .

## Discussion

This systematic review and meta-analysis explored factors associated with immunization coverage in Ethiopia. To our knowledge, this is the first evidence-based estimate of predictors of immunization coverage among 12–23-month-old children. Maternal educational status, paternal educational status, residence, place of delivery, family size less than four, maternal knowledge on age immunization to be completed, maternal knowledge on the immunization schedule, knowledge on the benefit of immunization, traveling time to the health facility, antenatal care, and TT vaccination was found to be significantly associated with immunization coverage in Ethiopia.

Our study found parental literacy to be a significant predictor of immunization coverage among children age 12–23 months. In this regard, we found that parents that had formal education were more likely to fully vaccinate their children compared to their counterpart. Our finding is consistent with other studies that revealed parents who attended formal education were more likely to provide all antigens to their children [54–60]. The current work is also consistent with a study done in Uganda that revealed 46% of mothers who had primary education fully vaccinated their children while 65% of those attended secondary education fully vaccinated their children. Thus as maternal education status increases the likelihood of children receiving all antigens increases [61]. Similarly, studies done in Asian countries like India, Vietnam, and Lebanon revealed formally educated mothers are more likely to complete routine immunization than those who did not attend school [62–65]. In contrast, the study done in Ghana revealed that maternal educational status was not significantly associated with full vaccination [55]. The possible reasons that parents with primary and higher education are more likely to utilize information education and communication (IEC) and understand the importance of immunization service [66].

Our study also found that location of residence played a great role in immunization completion among children. Children that live in urban areas were 1.57 times more likely to be fully vaccinated than those in rural areas. Earlier studies that explored the relationship between residence and immunization coverage reported mixed findings [54, 63, 64, 67] . Some studies found a strong relationship between urban residence and immunization while others did not [55, 68]. The possible explanation for this disparity could be inherent socio-economic variations that existed among different study settings. Rural Some settings might exist extremely disadvantaged than urban. Disparity in access to health facility and education are common themes which emerge in many studies conducted in urban-rural communities in many countries, including Ethiopia [69, 70].

Furthermore, the place of delivery was found to be a significant predictor of immunization coverage among children age 12–23 months. To this end, women who gave birth at the health facility were 1.86 times more likely to complete all required antigen to their children compared to women who gave birth at home. This finding is consistent with other findings conducted in many parts of the world [59, 62, 63, 71]. On the other hand, studies found home delivery as one of the factors for incomplete vaccination [55, 72, 73]. This could be explained by the fact that the first dose (BCG and OPV0) of routine immunization is provided immediately after delivery, which may increase maternal awareness and motivate mothers to complete the sequential doses [74]. Besides, home delivery if taken as a proxy for women’s decision-making autonomy on child’s healthcare may reflect its negative influence on child’s immunization coverage [75, 76]. Another study also showed that women that had decision-making autonomy were more likely to utilize both institutional delivery, postnatal follow-ups, and child healthcare services [77].

The present study also found that family size was much associated with immunization coverage. Households that had less than four children were more likely to vaccinate their children compared to their counterparts. This finding is in line with studies conducted in Australia [67], India [78], Lebanon [65], Ghana [55] and Brazil [79]. In contrast, the study conducted in Zimbabwe revealed a weak association between household size and immunization coverage [80]. The association between family size and immunization could be explained by the fact that a large family size is likely to hinder maternal capabilities to extend more care to the younger children as well as her mobility to get access to immunization services.

Moreover, in this systematic review and meta-analysis we observed that mothers who are aware of immunization schedule and age the child to complete the immunization program were more likely to complete routine vaccination compared to their counterparts. This finding is in line with a study conducted in Nepal that revealed mothers who had poor knowledge of immunization schedule and age to complete the immunization were four times more likely to complete the immunization program for their children than their counterparts [81]. Likewise, a study done in India showed that 93.5% of incomplete or partial vaccination was due to lack of knowledge on immunization schedule [82]. This might be due to the fact that mothers who are aware of the immunization schedule were most likely to be well-informed on advantage of immunization and age to complete immunization services, though our findings found no association between knowledge on the benefit of immunization and immunization coverage.

In Ethiopia, distance to the health facility is a major challenge leading to less access to the health services [8]. Our study found traveling time more than a 60 min walk to be a negative contributing factor to immunization coverage. Women whose walk to the health facility was less than or equal to 60 min were more likely to vaccinate their children compared to those with a walk above 60 min. This finding is supported by studies done in different parts of the globe [56, 68, 72, 83].

We also found that maternal service utilization to be a predictor of child immunization coverage. Mothers who followed ANC were more likely to complete vaccination for their children compared to their counterparts. This finding is in line with studies conducted in Pakistan [73], Myanmar [84] Indonesia [78], Senegal [59] India [85], and Philippines [86], and a study done in 46 low and middle income countries (LMIC) [87]. Mothers who are following both ANC and PNC are more likely to interact with health care providers and exposed to information about maternal and newborn health services [78]. Similarly, mothers who took TT vaccination were almost five times more likely to complete vaccination for their children compared to those who were not vaccinated. This finding is comparable with a study done in Myanmar [84] that revealed an association between TT vaccination and immunization coverage among children 12–23 months. Maternal service utilization such as ANC and TT vaccinations are found to be proxy indicators that enhanced 19 and 13% immunization coverage among children aged 12–23 months respectively [88]. Similarly, institutional delivery [89] and a decision made during pregnancy to vaccinate the child also increases the chance of full vaccination [90].

### Strengths

We registred the protocol in Prospero and strictly followed PRISMA guidelines. We have also done a comprehensive literature search and included more factors for this study.

### Limitation

The current work is not without limitations. First, we reviewed only observational studies that cannot be used to infer cause and effect relationship. Community-based studies are also prone to recall bias for infant’s vaccination status. Restriction of our search to only English language articles limited the number of articles included in this systematic review and meta-analysis. High heterogeneity was observed from included factors in spite of using the random-effect model. The current work also did not include grey literature/unpublished literature and the potential for publication bias. No study found from some regions of Ethiopia like Benshagul Gumuz.

## Conclusion

Our findings showed that full immunization among 12–23 months old children are affected by the individual, community, and health service delivery level factors such as literacy, residence, awareness, family size, maternal health services utilization, and proximity of the health facilities were factors associated with full immunization among 12–23 months old children. This implies that there is a need for primary health service expansion and health education to “hard to reach areas” to improve immunization coverage for children aged 12–23 months.

## Supplementary Information

**Additional file 1: Appendix I.** Searching strategy.

**Additional file 2: Appendix II.** Data extraction checklist.

**Additional file 3: Appendix III.** JBI critical appraisal for case-control.

**Additional file 4: Appendix IV.** JBI critical appraisal tools cross-sectional.

**Additional file 5: Appendices IV.** Newcastle-Ottawa Scale adapted for cross-sectional studies.

## Data Availability

All data generated or analyzed during this systematic review and meta-analysis is included in this article.

## Abbreviations

BCG: Bacillus Calmette Guerin
CI: Confidence Interval
DHS: Demographic Health Survey
DPT: Diphtheria Pertussis Tetanus
EDHS: Ethiopia Demographic Health Survey
EPI: Expanded Program of Immunization
HMIS: Health Management Information System
IRIS: Institutional Repository for Information Sharing
JBI: Joanna Briggs Institute
LMIC: Low and Middle Income Countries
PNC: Postnatal Care
PRISMA: Preferred Reporting Items of Systematic Reviews and Meta-Analysis
SDG: Sustainable Development Goals
TT: Tetanus Toxoid
VPD: Vaccine Preventable Diseases
WHO: World Health organization
