# Supplementary material for: Predictors of immunization coverage among 12–23 month old children in Ethiopia: systematic review and meta-analysis
Source: BMC Public Health. 2020 Nov 26;20:1803. doi: 10.1186/s12889-020-09890-0 (PMC7689978; doi:10.1186/s12889-020-09890-0)
Supplement: Supplementary file 1 — Additional file 1: Appendix I. Searching strategy. [file 12889_2020_9890_MOESM1_ESM.docx]

Appendix I. Searching strategy

*“Coverage, Vaccination OR Coverage, Vaccination OR Vaccination Coverage OR Immunization Coverage OR Coverage, Immunization OR Coverages, Immunization OR Immunization Coverages OR Vaccinations OR Immunization, Active OR Active Immunization OR Active Immunizations OR Immunizations, Active AND Children AND Epidemiologic Factor OR Factor, Epidemiologic, Epidemiologic Determinant OR Determinant, Epidemiologic OR Determinants, Epidemiologic OR Epidemiologic Determinants OR Factors, Epidemiologic AND Ethiopia” Filters: Free full text; AND* (“2009/01/01”[PDat]: “2020/01/01”[PDat]) AND Humans [Mesh].
