# Supplementary material for: Predictors of immunization coverage among 12–23 month old children in Ethiopia: systematic review and meta-analysis
Source: BMC Public Health. 2020 Nov 26;20:1803. doi: 10.1186/s12889-020-09890-0 (PMC7689978; doi:10.1186/s12889-020-09890-0)
Supplement: Supplementary file 2 — Additional file 2: Appendix II. Data extraction checklist. [file 12889_2020_9890_MOESM2_ESM.docx]

**Appendix II. Data extraction checklist**

Author ____________________________

Year of publication __________________

Country ___________________________

Study area__________________________

Aim / scope ________________________

Population _________________________

Methodology/Method ________________

| intervention | | |  | without intervention | |
| --- | --- | --- | --- | --- | --- |
| s.no | No of events | Sample size |  | No event | Sample size |
| 1 |  |  |  |  |  |
| 2 |  |  |  |  |  |
| 3 |  |  |  |  |  |

Summary of result and their interpretation _______________________________________

Author’s conclusion __________________________________________________________
