# Supplementary figures and images for: Predictors of immunization coverage among 12–23 month old children in Ethiopia: systematic review and meta-analysis
Source: BMC Public Health. 2020 Nov 26;20:1803. doi: 10.1186/s12889-020-09890-0 (PMC7689978; doi:10.1186/s12889-020-09890-0)

Appendix III JBI critical appraisal for case-control.


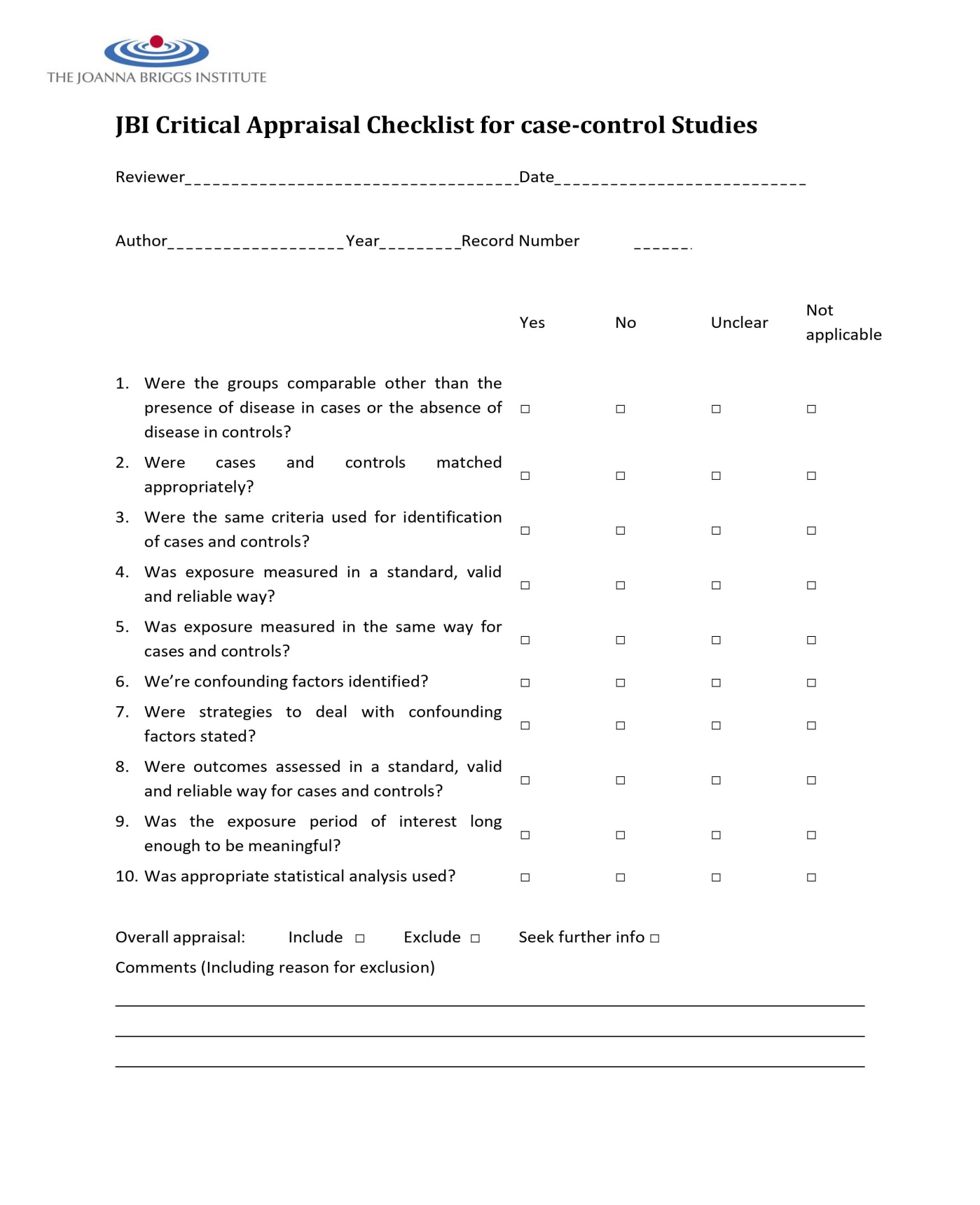

Supplement: Supplementary file 3 — Additional file 3: Appendix III. JBI critical appraisal for case-control. [file 12889_2020_9890_MOESM3_ESM.docx]

**Appendix IVJBI critical appraisal tools cross-sectional**


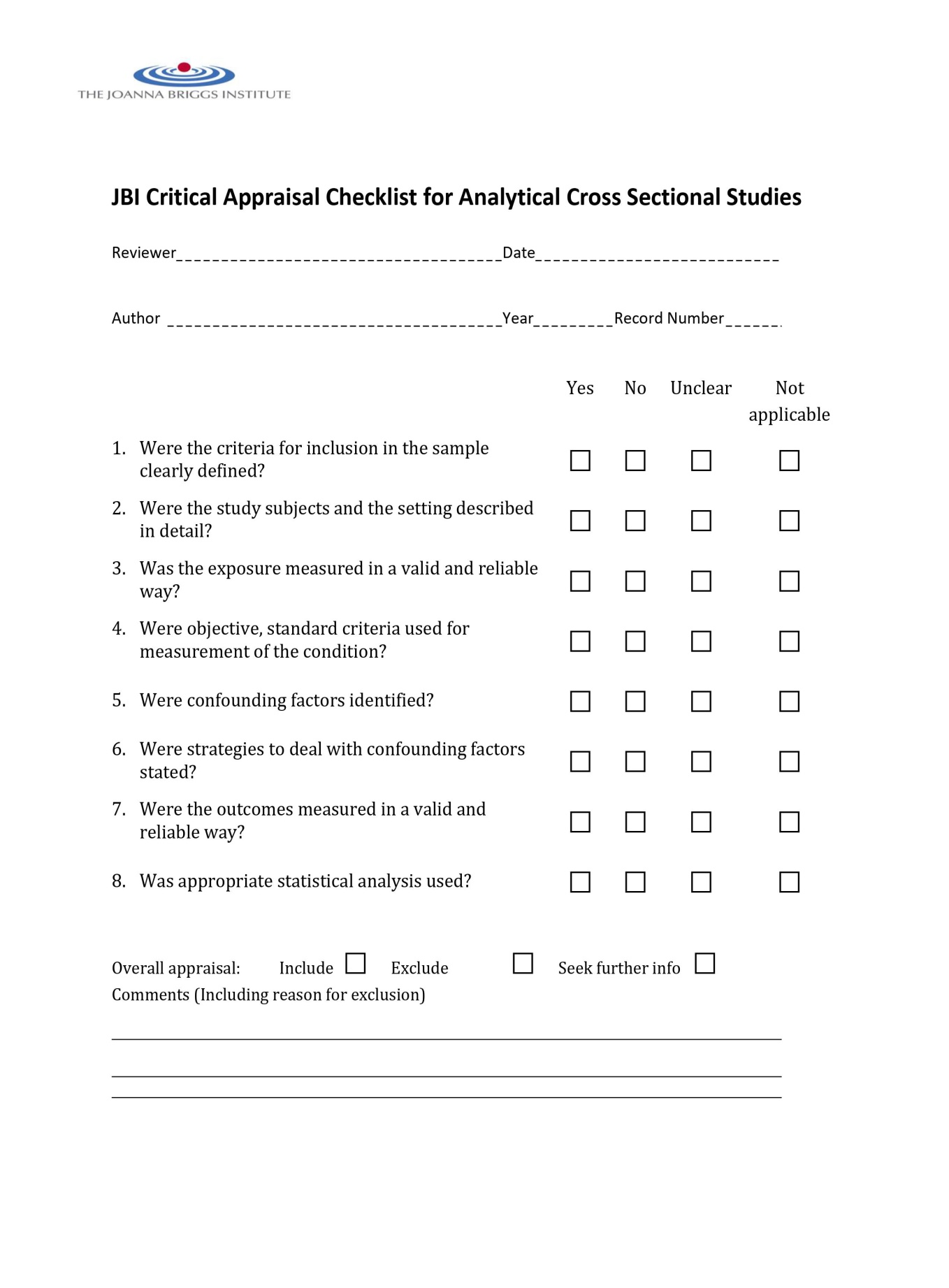

Supplement: Supplementary file 4 — Additional file 4: Appendix IV. JBI critical appraisal tools cross-sectional. [file 12889_2020_9890_MOESM4_ESM.docx]
